# Fleas from the Silk Road in Central Asia: identification of *Ctenocephalides canis* and *Ctenocephalides orientis* on owned dogs in Uzbekistan using molecular identification and geometric morphometrics

**DOI:** 10.1186/s13071-022-05477-3

**Published:** 2022-09-29

**Authors:** Georgiana Deak, Alisher Safarov, Xi Carria Xie, Runting Wang, Andrei Daniel Mihalca, Jan Šlapeta

**Affiliations:** 1 Department of Parasitology and Parasitic Diseases, Faculty of Veterinary Medicine, University of Agricultural Sciences and Veterinary Medicine of Cluj-Napoca, Calea Mănăștur 3-5, 400372 Cluj-Napoca, Romania; 2 State Committee of Veterinary and Livestock Development of the Republic of Uzbekistan, Tashkent, Uzbekistan; 3 Sydney School of Veterinary Science, Faculty of Science, University of Sydney, Sydney, NSW Australia

**Keywords:** *Ctenocephalides*, Dogs, Fleas, Uzbekistan, *cox1*, Geometric morphometrics

## Abstract

**Background:**

The Silk Road connected the East and West for over 1500 years. Countries in Central Asia are valuable in addressing the hypothesis that parasites on domestic animals were introduced along the Silk Road. Adult fleas are obligate parasites, having worldwide distribution. In dogs, *Ctenocephalides canis*, *C. felis* and *C. orientis* are the most common species identified. The distribution of the Oriental cat flea, *C. orientis*, is restricted to southeast Asia. The purpose of this study was to determine the diversity of dog fleas from Uzbekistan, a country in Central Asia, with particular reference to *C. orientis*.

**Methods:**

Fleas were collected from 77 dogs from 5 locations in Uzbekistan. The *cox1* gene sequences from *Ctenocephalides* spp. were compared to global collection of *Ctenocephalides cox1* haplotypes. Landmark-based geometric morphometrics have been applied to the head and curvature to compare *C. canis* and *C. canis* using canonical variate analysis and discriminant function analysis*.*

**Results:**

Overall, 199 fleas were collected and identified as *C. canis* (*n* = 115, 58%), *C. orientis* (*n* = 53, 27%) and *Pulex irritans* (*n* = 22, 11%). None of the fleas were *C. felis*. All *Ctenocephalides* spp. fleas were subject to *cox1* amplification and 95% (166/175) yielded DNA sequence. There were 25 *cox1* haplotypes; 14 (22/25, 88%) were *C. canis cox1* haplotypes and 3 (3/25, 12%) were *C. orientis cox1* haplotypes. Molecular analysis confirmed the absence of *C. felis*. Four (4/22) and one (1/3) *cox1* haplotypes were identical to *cox1* haplotypes belonging to *C. canis* and *C. orientis cox1* haplotypes identified elsewhere, respectively. Overall morphometric analysis confirmed significant differences between the head shape of *C. canis* and *C. orientis* and improved four–fivefold the species identification compared to traditional morphological key.

**Conclusion:**

We report for the first time the presence of *C. orientis* in Uzbekistan. Differentiation of *C. orientis* from *C. canis* and *C. felis* remains difficult in regions where these species coexist. Studies in Central and Southeast Asia should confirm species identity using *cox1* locus to enable retracing of the distribution of the *Ctenocephalides* in Asia. The presence of *C. orientis* suggests that this species may have been introduced from the east along the ancient Silk Road.

**Graphical Abstract:**

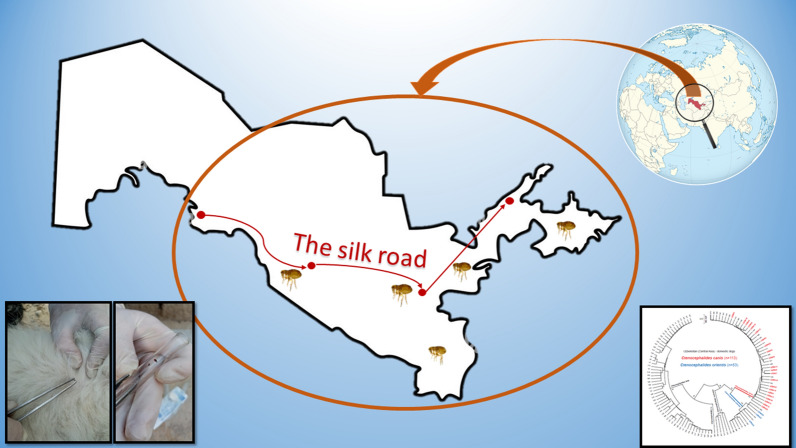

**Supplementary Information:**

The online version contains supplementary material available at 10.1186/s13071-022-05477-3.

## Background

The order Siphonaptera (fleas) is a highly specialized order of holometabolous parasitic insects, comprising more than 200 genera with over 2500 recognized species. Adult fleas are obligate external parasites of birds and mammals, having a worldwide distribution, including cold areas (Antarctica). They can inhabit a large range of hosts and habitats [1–3]. In domestic carnivores, namely dogs and cats, fleas are common ectoparasites, responsible for up to 50% of all dermatological cases [4]. Responsible owners spend hundreds of dollars per year on ectoparasitic products against fleas [5]. Due to urbanization, specific services of some domestic dogs (hunting, security and shepherd), their contact with wild carnivores has significantly increased and represents an important spill-over of parasites, which could be a threat to human health due to the potential transmission of zoonotic pathogens [6, 7].

Three species of fleas are known to be the most common ones identified in domestic dogs and cats worldwide, apart from Antarctica, namely *Ctenocephalides canis* (Curtis, 1826), *Ctenocephalides felis* (Bouché, 1835) and *Ctenocephalides orientis* (Jordan, 1925) [8]. In Australia, Central and Southwestern Europe, South and Southeast Asia, the dominant species in dogs is *C. felis* [8–13]. Interestingly, in dogs of several Eastern Europe countries, a more common infestation by *C. canis* and even *Pulex irritans* Linnaeus, 1758, occurs [14]. The dog flea, *C. canis*, was dominant also in dogs from South Korea [15], while in India the most common species detected in dogs was *C. orientis*, a species previously associated with other animal species, such as ruminants [8, 16, 17]. The differentiation of *C. canis* from *C. orientis* requires fine morphological observation or molecular confirmation, and it is likely that past studies from Asia and Southeast Asia may have misidentified *C. orientis* as *C. canis* [8, 16]. Even though dog fleas are of great veterinary and human-health interest, there are very few to zero studies regarding the flea species diversity on companion animals and flea-borne pathogen occurrence in countries of Central Asia [18, 19].

The only study on the flea fauna of dogs in Uzbekistan revealed the presence of *C. canis* and *C. felis*. However, details about their prevalence or other epidemiological characteristics are not given [19]. The Republic of Uzbekistan has a wide diversity of wild and domestic carnivores, including about 2.5 million domestic dogs kept as pets or for security reasons [20, 21] (Information from the State Committee for Veterinary and Livestock Development of the Republic of Uzbekistan as of January 1, 2022). Traditional animal husbandry is still practiced in this country, favoring a close contact of domestic dogs with wildlife [22].

The aims of the present study were to determine the fleas’ identity using a combination of traditional morphological identification, molecular identification and landmark-based geometric morphometric analysis.

## Methods

The present study included a total of 77 dogs which were sampled over a period of 3 months during the winter season (December 2020 to February 2021) in Uzbekistan, Central Asia. The dogs were from the following five cities along the former Silk Road trade route between the East and the West: Jizzax, Buxoro, Farg’ona, Samarkand and Surkxandaryo. The included dogs were housed outside, in rural areas, and served as security dogs for the protection of farm animals (cattle, sheep, poultry) from wild predators (wolves, jackals, foxes), being kept as free ranging. The owners were questioned about the ectoparasitic treatments, age and sex of each examined animal. The study was approved by the Animal Care Organization of the State Committee for Ecology and Environmental Protection.

The studied population of dogs was characterized according to age, gender (males and females) and geographical location. The age was recorded in months and based on this criterion, dogs were divided into two groups: juveniles (0 to 12 months) and adults (over 12 months). Fleas were opportunistically manually collected using fine entomological tweezers and by combing. The collection of ectoparasites was not related to a specific protocol and some of the specimens might have been missed.

Collected specimens were placed and stored in absolute ethanol in 2 ml labeled tubes. The preserved samples were sent to the Department of Parasitology and Parasitic Diseases of the University of Agriculture and Veterinary Medicine of Cluj-Napoca where genomic DNA was individually extracted with the preservation of the exoskeletons as previously described [11, 23] using a commercially available kit (Isolate II Genomic DNA, Bioline, UK). The remaining exoskeletons were further digested in 10% KOH for 2–3 h, washed in 10% acetic acid for 30 min and then dehydrated using a series of ethanol washes (25%, 50%, 75%) for 30 min in each concentration. Before the final step, the fleas were kept in clove oil for 45–60 min and slide-mounted in natural Canada Balm (Roth, Germany). The slides were dried at 55 ˚C for about 4–6 weeks to facilitate the elimination of the air bubbles. The morphological identification was done to species level using specific descriptions and keys [8, 24]. Morphological identification of *Ctenocephalides* spp. was aided with subjective observation of the aspect of the angle of the cephalic profile and on the number and orientation of the setae as previously described [8].

Each specimen was photographed using a digital camera attached to a microscope, and images were used to build ‘tps’ files in tpsUtil V1.81 and tpsDig V2.32 [http://www.sbmorphometrics.org/soft-utility.html]. We identified five landmarks of the flea head with one curve that was resampled into ten landmarks (2 matched existing landmarks); a final dataset included 13 landmarks across the head of each flea for geometric morphometric analysis in MorphoJ V1.07a [https://morphometrics.uk/MorphoJ_page.html] [25]. Raw landmark coordinates were aligned and superimposed using Procrustes fit function to remove variation due to differences in scale, position and orientation from the coordinates. Head shape variation was analyzed using MorphoJ software [25]. Canonical variate analysis (CVA) was used to determine the most important feature as a possible discriminator between groups (sex or/and species). The statistical significance of pairwise differences in mean shapes was analyzed using permutation tests (10,000 rounds) with Mahalanobis distances and Procrustes distances. Additionally, a cross-validation test in discriminant function analysis (DFA) was used to assess the accuracy of classification based on Mahalanobis distances in a permutation test with 10,000 rounds using MorphoJ software [25].

Molecular identification for all *Ctenocephalides* spp. fleas followed previously established protocol [8]. An aliquot of the extracted DNA (30 µl) was sent to the Sydney School of Veterinary Science at the University of Sydney, Australia, for amplification of partial cytochrome c oxidase subunit 1 (*cox1*) and DNA sequencing. The partial *cox1* (550–650 bp) fragment from individual flea DNA (*n* = 175) was amplified using a combination of LCO1490 (5′-GGT CAA CAA ATC ATA AAG ATA TTG G-3′) (Folmer et al. 1994) and/or Cff-F [S0367] (5′-AGA ATT AGG TCA ACC AGG A-3′) with Cff-R [S0368] (5′-GAA GGG TCA AAG AAT GAT GT-3′) [11] primers. Amplification (30 μl) was performed with MyTaq Red Mix (BioLine), primers and 2 μl of DNA in nuclease-free water in a T100 cycler (Bio-Rad, Australia). The cycling condition included an initial denaturation at 95 °C for 1 min followed by 35 cycles of 95 °C for 15 s, 55 °C for 15 s and 72 °C for 10 s, and the amplification was finished with a final elongation for 5 min at 72 °C. All reactions were run with a negative control that included sterile PCR water instead of DNA. The PCR products of the expected size (550–650 bp) were sequenced at Macrogen Inc. (Seoul, Korea). The raw bidirectional Sanger sequence chromatographs were assembled and compared to a reference dataset of 90 haplotypes (h1-h90) sensu [8] using CLC Main Workbench 21 (CLC bio, Qiagen, Australia). Newly obtained unique sequences were appended to the *Ctenocephalides cox1* haplotype alignment from Lawrence et al. [8] and pairwise distances and phylogenetic tree using Kimura-2 (K2) distances constructed using Minimum Evolution (ME) method with bootstrap support (1000 replicates) in MEGA 11 [26].

The data were incorporated in a Microsoft Excel sheet, and proportion, standard error, prevalence and 95% confidence intervals (CI) were calculated for each of the obtained results. The descriptive data are available in the additional file in the “stats” sheet. The distribution map based on the presence of flea species was generated using ArcMap 10.6.1.

## Results

We examined 77 dogs from Uzbekistan in Central Asia. The dogs originated from Jizzax (*n* = 24), Buxoro (*n* = 12), Farg’ona (*n* = 19), Samarkand (*n* = 20) and Surkxandaryo (*n* = 2). The age of the tested dogs ranged from 6 months to 2 years and 2 months, with an average age of 10.65 ± 5.26 months. Approximately equal numbers of dogs were females (*n *= 38) and males (*n* = 39). Dog owners reported that none had ever been treated with antiparasitic products.

On physical examination, 56% (43/77, 95% CI 45–67%) of dogs were found to be infested with fleas. Based on the dogs age, more infested dogs were < 1 year old (*n* = 33, 59% 95% CI 46–72%) compared to those > 1 year old (*n* = 8, 38%, 95% CI 17%-59%).

**Fig. 1:**
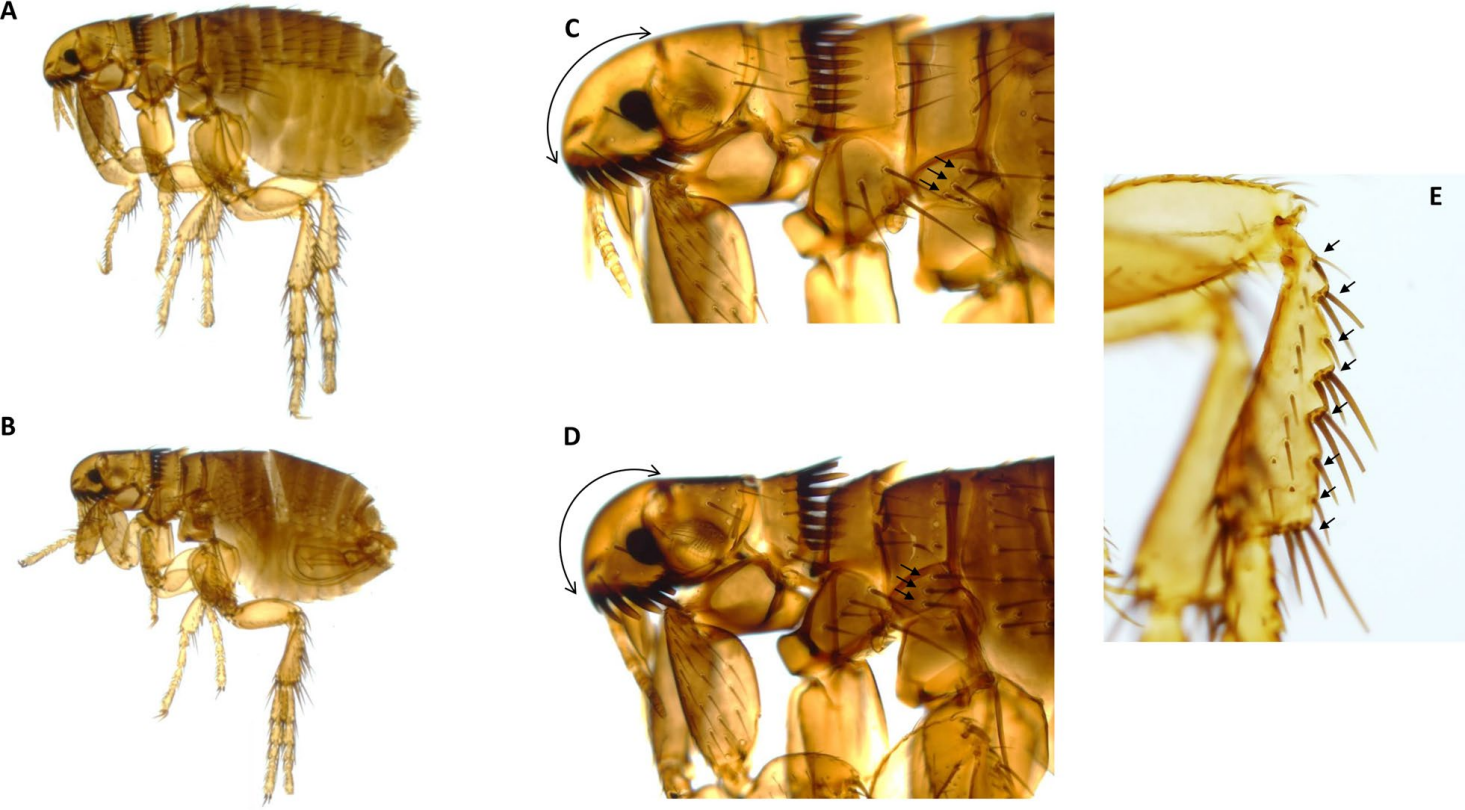
Morphological aspects of *C. canis* specimens collected from dogs in Uzbekistan. **A** General aspect of a female *C. canis*. **B** General aspect of a male *C. canis*. **C** The aspect of the angle of the cephalic profile of a female. Note the number (3) and the orientation of the setae in the metanotal area (marked with black arrows). **D** The aspect of the angle of the cephalic profile of a male. Note the number (3) and the orientation of the setae in the metanotal area (marked with black arrows). **E** Morphology of hind tibia: the numbers of setae (8) with notches are marked with black arrows

**Fig. 2:**
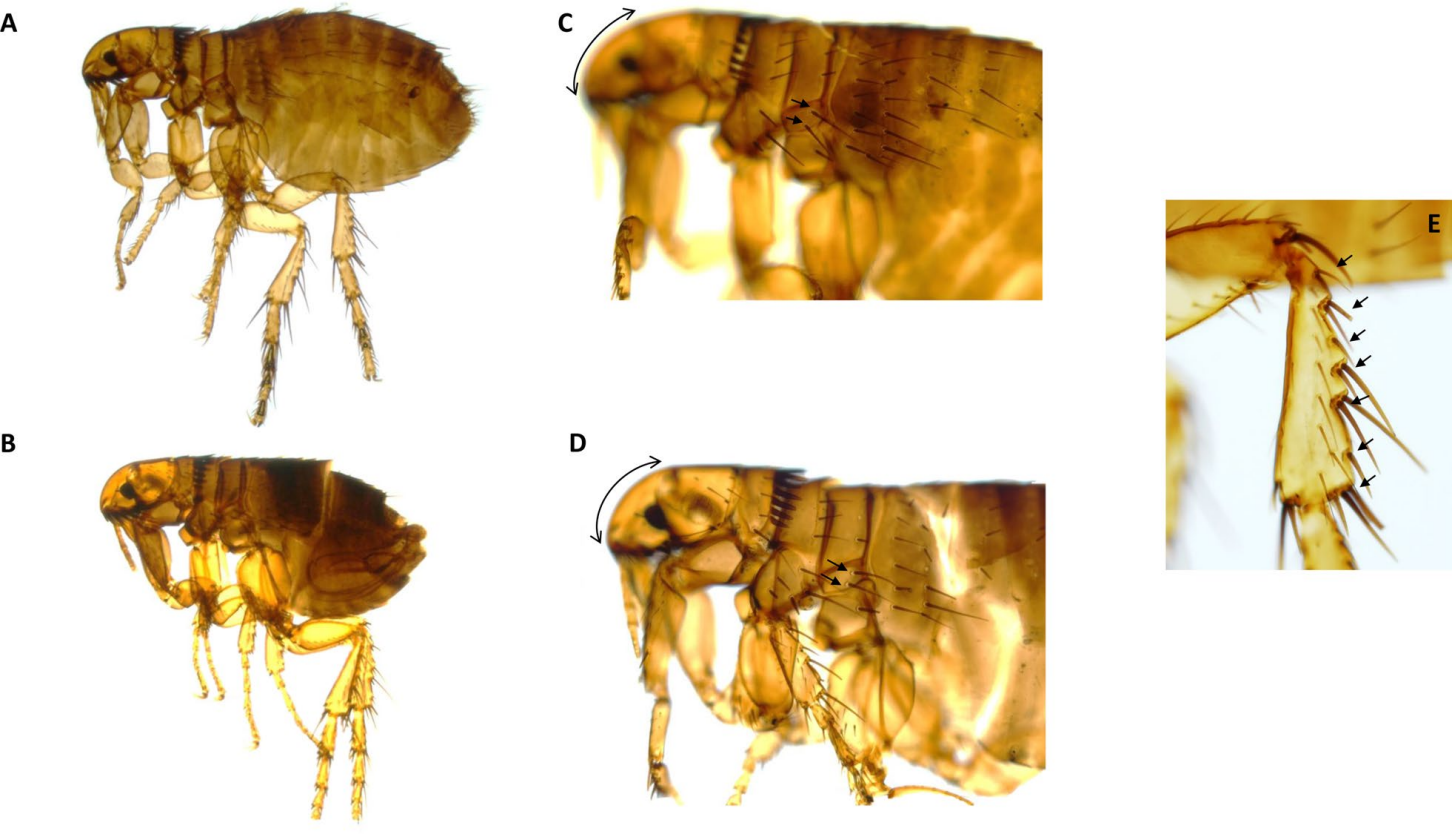
Morphological aspects of *C. orientis* specimens collected from dogs in Uzbekistan. **A** General aspect of a female *C. orientis*. **B** General aspect of a male *C. orientis*. **C** The aspect of the angle of the cephalic profile of a female. Note the number and the orientation of the setae (2) in the metanotal area (marked with black arrows). **D** The aspect of the angle of the cephalic profile of a male. Note the number and the orientation of the setae (2) in the metanotal area (marked with black arrows). **E** Morphology of hind tibia: the number of setae (7) with notches are marked with black arrows

Overall, 197 fleas were collected and morphologically identified as *C. canis* (*n* = 94) (Fig. 1), *C. orientis* (*n* = 61), *Ctenocephalides* spp. (*n* = 19) and *P. irritans* (*n* = 22, 11%) (Fig. 2). None of the examined fleas were identified as *C. felis*. Nineteen specimens were classified as *Ctenocephalides* spp. without species identification due to the damage to their exoskeleton and/or ambiguous characteristics that precluded species identification.

**Fig. 3:**
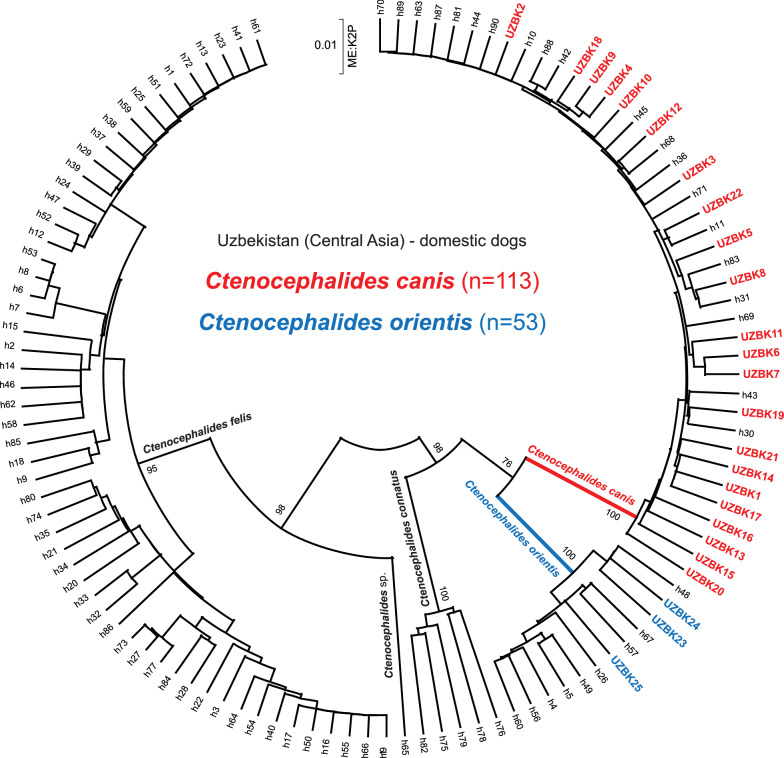
Phylogenetic relationships of *cox1* haplotypes of *Ctenocephalides canis* and *C. orientis* from Uzbekistan. The phylogenetic tree used all 17 unique haplotypes from *Ctenocephalides* fleas from Uzbekistan (UZBK1-17) together with 90 unique haplotypes (h1-h90; [8]). The tree was inferred using Minimum Evolution (ME) and the Kimura-2 distances. Bootstrap support values (from 1000 replicates) are shown only for the major branches leading to *Ctenocephalides* spp. Branches leading to *cox1* sequences from *C. canis* and *C. orientis* are color coded

All fleas belonging to the genus *Ctenocephalides* were subject to *cox*1 amplification, and 95% (166/175) yielded DNA sequence. There were 25 cox1 haplotypes (UZBK1-25) among the *Ctenocephalides* DNA sequences; 14 (88%, 22/25) were *C. canis cox*1 haplotypes [UZBK1 (*n* = 56), UZBK2 (*n* = 20), UZBK3 (*n* = 7), UZBK4 (*n* = 2), UZBK5 (*n* = 2), UZBK6 (*n* = 1), UZBK7 (*n* = 1), UZBK8 (*n* = 7), UZBK9 (*n* = 1), UZBK10 (*n* = 1), UZBK11 (*n* = 2), UZBK12 (*n* = 1), UZBK13 (*n* = 1), UZBK14 (*n* = 1), UZBK15 (*n* = 1), UZBK16 (*n* = 1), UZBK17 (*n* = 1), UZBK18 (*n* = 2), UZBK19 (*n* = 1), UZBK20 (*n* = 2), UZBK21 (*n* = 1), UZBK22 (*n* = 1)] and 3 (12%, 3/25) were *C. orientis* [UZBK23 (*n* = 25), UZBK24 (*n* = 18) and UZBK25 (*n* = 10)]. Molecular analysis confirmed absence of *C. felis*. Phylogenetic analysis confirmed close relationship of the UZBK *cox*1 haplotypes with those previously identified in [8] (Fig. 3). Using Kimura-2 distance the maximum interspecies distance for the *cox*1 locus was 0.012 for *C. canis*, 0.064 for *C. orientis* and 0.092 for *C. felis*. Only four (4/22) *cox*1 haplotypes were identical to those *cox1* haplotypes belonging to *C. canis* reported by Lawrence et al. [8]; UZBK2 = h10, UZBK8 = h31, UZBK19 = h30 and UZBK22 = h11. One out of three (1/3) *C. orientis cox*1 haplotypes were identical to previously identified haplotypes in Lawrence et al. [8]; UZBK23 = h48.

**Fig. 4:**
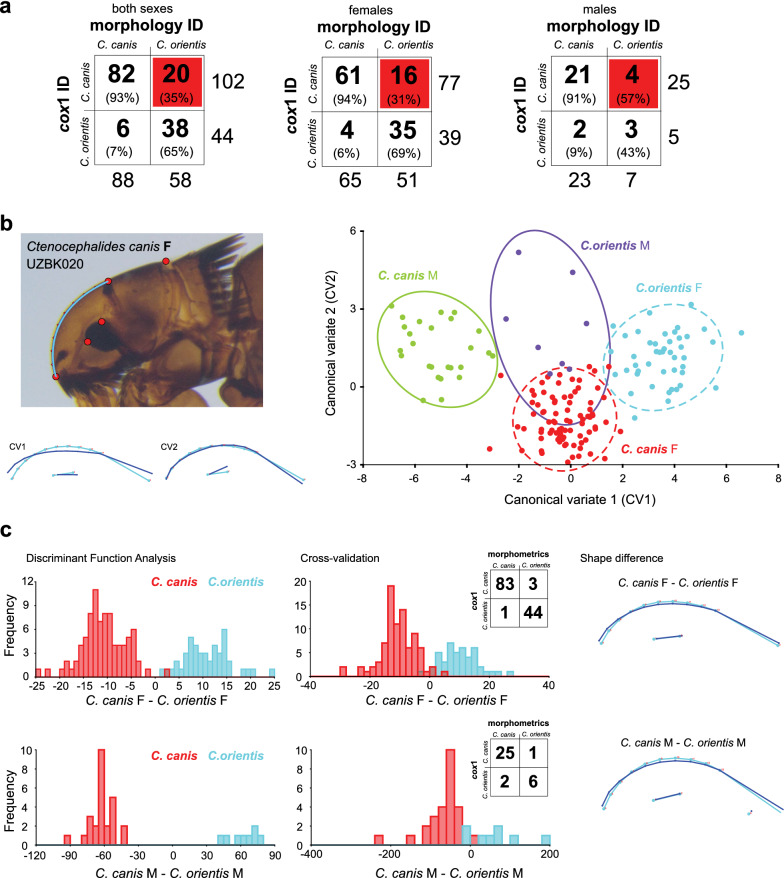
*Ctenocephalides* species identification based on molecular, morphological and geometric morphometrics. **a** Contingency tables summarizing identification of *Ctenocephalides* species based on traditional morphological key (morphology ID) and molecular approach using *cox1* (*cox*1 ID). The percentages represent values for traditional morphological key identification against the reference identification that is considered here the molecular identification using cox1 sequence. Only specimens that were fully identified using both methods are included. The red squared cell indicates percentage of misidentification of traditional morphological key to identify *C. orientis*. **b** Flea head of female *Ctenocephalides canis* showing the five plotted landmarks and one curve that was resamples into ten landmarks. Scatter plot showing the variation in shape of flea head along the first two canonical variate (CV1 and CV2) axes with 90% confidence ellipses (dotted like: females, continuous line: males). Each species (*C. canis* and *C. orientis*) and sex (F: female; M: male) were color coded to show separation from each other. Wireframe grids illustrate the shape changes from overall mean along CV1 and CV2 axes and directions of changes based on the superimposed wireframes; red dots indicate the locations of the landmarks. **c** Discriminant function analysis examining separation of a pair of between *Ctenocephalides* species separated according to their sex; female and male presented separately. For each, a histogram with the values of the discriminant scores for the original data and another histogram with the scores for the leave-one-out cross-validation is shown followed by a diagram with the shape differences between the two groups using wireframe. The inset within the leave-one-out cross-validation shows a contingency table summarizing classification according to discriminarory function analysis (morphometrocs) compared to molecular identification (*cox*1)

Comparing morphological with molecular identification of *Ctenocephalides* spp., we noted relatively good agreement for morphological identification of *C. canis* with 91–93% specimens correctly identified considering *cox*1 identified the species (Fig. 4a). Morphological identification appears to be more problematic for *C. orientis* based on morphology with overall 65% specimen identified correctly, and identification of females was marginally better reaching 69% correctness but for males reached only 43%. Misidentification was frequent for *C. orientis*, assuming morphological males of *C. orientis* were *C. canis* (57%, 4/7) based on *cox*1; morphological females of *C. orientis* were *C. canis* (31%, 16/51) (Fig. 4a, highlighted in red). Molecular identification unambiguously identified all 19 *Ctenocephalides* spp. that we were unable to assign to a species morphologically, 10 belonged to *C. canis* (1 male, 9 females), and 9 belonged to *C. orientis* (3 males, 6 females) based on *cox1*. The noted uncertainty revolving around morphological *Ctenocephalides* species identification was caused by key characteristics that traditionally differentiate the two species appearing variable. In particular, the numbers of setae-bearing notches on the hind tibia for *C. canis* and *C. canis* are eight and seven, respectively [8]. In the present dataset, there were specimens that had 6–9 setae bearing notches on hind tibia in combination with other characteristics made the species identification challenging. With the accessibility of the *cox*1 identification and retained exoskeletons, we considered using geometric morphometrics to review whether the head shape aligns with *cox*1 identity, and thus if it is species specific, and potentially improves morphological identification (Additional file 1).

Head landmarks were collected for 165 specimens of *Ctenocephalides* spp. Based on *cox*1, there were 86 females of *C. canis*, 26 males of *C. canis*, 45 females of *C. orientis* and 8 males of *C. orientis* (Fig. 4b). There was a significant difference between the shape of the sexes and species (Table 1). Using canonical variate analysis (CVA), there were three canonical variates (CVs) that accounted for 100% variance in the datasets. First, two variates accounted for 97.3% of the variance (CV1: 79.3% and CV2: 18.8%) (Fig. 4b). Using a scatter plot and grouping based on *Ctenocephalides* species and sex showed clear clusters, and all groups were significantly different (*P* < 0.001) from each other using permutation tests (10,000 permutation rounds) for Mahalanobis and Procrustes distances among groups. We then restricted the dataset to only female and male specimens to undertake discriminant function analysis (DFA). Discriminant function between females or males of *C. canis* and *C. orientis* demonstrated a significant difference between pairs of groups of Procrustes and Mahalanobis distances (*P* < 0.0001 based on 1000 permutations). Reliability of the discrimination was then assessed by leave-one-out cross-validation showing that three (6%, 3/47) female *C. canis* have been morphologically classified as *C. orientis*, and one (1%, 1/84) female *C. orientis* has been classified morphologically as *C. canis.* For males, the leave-one-out cross-validation showed one (14%, 1/7) *C. canis* was classified morphologically as *C. orientis*, and two (7%, 2/27) *C. orientis* were classified as *C. canis* (Fig. 4c)*.* Overall, the geometric morphometrics decreased the misclassification of both species and reduced the high misclassification of *C. orientis* females from 31 to 6% (5.2-fold improvement) and for males of *C. orientis* from 57 to 14% (4.1-fold improvement) misidentifications, if only head shape is considered, using geometric morphometrics (Fig. 4a, c).

**Table 1:** Summary of the Procrustes ANOVA using shape for *Ctenocephalides canis* and *C. orientis*

Effect	SS	MS	Df	F	*P*-value (param.)
Flea sex	0.12271	0.00558	22	38.49	< 0.0001
Flea species	0.09495	0.00432	22	29.78	< 0.0001
Flea sex * species	0.00319	0.00015	22	2.88	< 0.0001
Residual	0.17843	0.00005	3542		

F statistics and parametric P-values for each of the effects in the ANOVA

*SS* sums of squares, *MS* mean squares, *df* degrees of freedom

*Significantly different

**Fig. 5:**
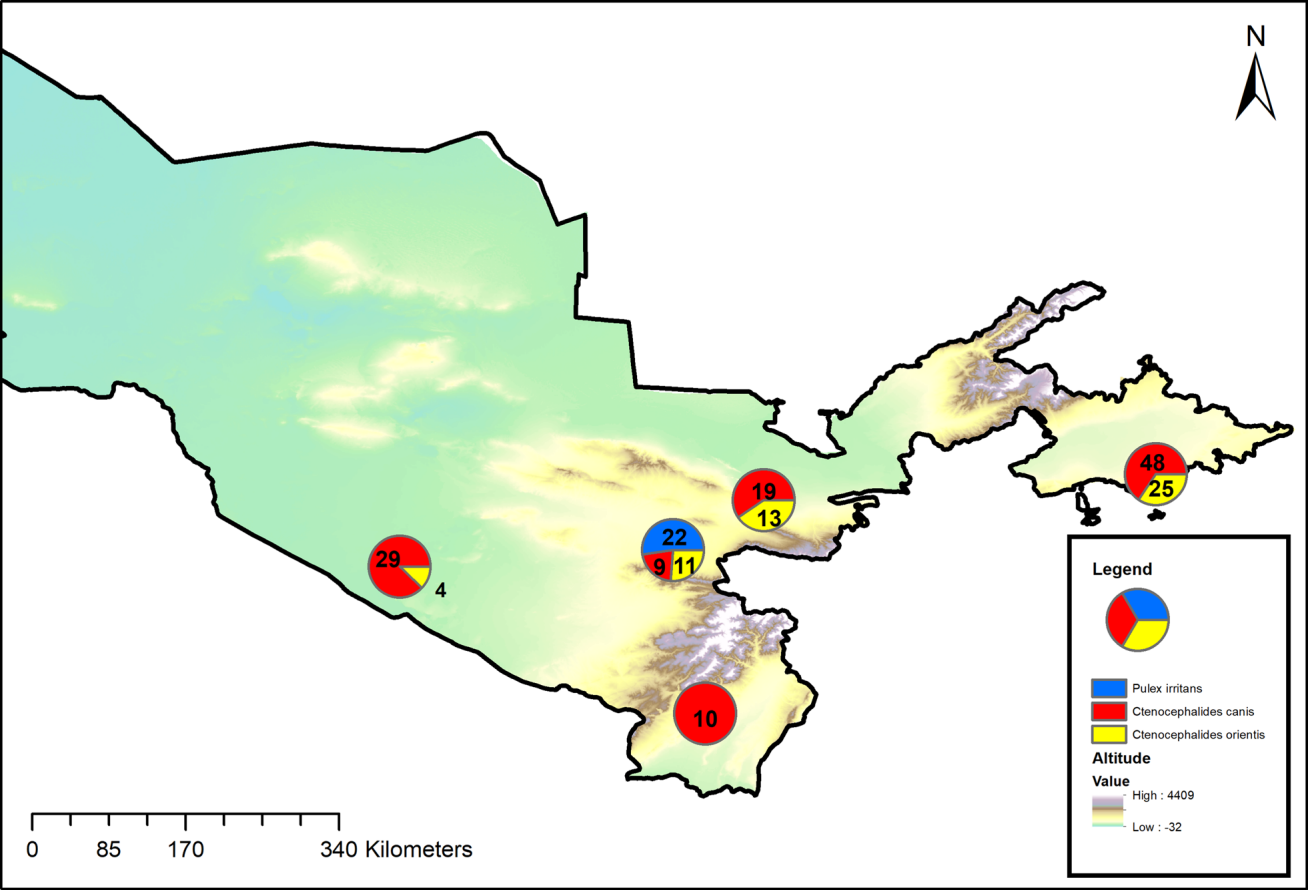
Map showing the geographical distribution of the identified flea species related to the altitude. Pie charts illustrate the proportions of identified fleas for each geographical location and the number of fleas identified for each species

The only flea recorded in all five sampling locations was *C. canis*. A total of 88 *C. canis* male specimens and 26 female specimens were collected (sex ratio female: male = 0.31). The sex was not determined for one of the identified specimens because of the degradation status of the posterior part. The second most abundant flea was *C. orientis* identified on dogs from four locations: Jizzax, Buxoro, Farg’ona and Samarkand. The sex ratio for *C. orientis* between female (*n* = 45) and male (*n* = 8) fleas was 5.62. *Pulex irritans* were identified only on dogs from Samarkand with a sex ratio between females (*n* = 20) and two males (*n* = 2) of 10. The fleas for which the species was not identified were eight females and one male (Table 2). The geographical distribution of the flea species identified in domestic dogs is presented in (Fig. 5). The complete morphological identification and all the details about the sex, age and locations of the included animals and the results of the fleas’ identification are available in the supplementary material.

**Table 2:** Number of animals examined from each geographical location of Uzbekistan and the flea species identified in each of the locations

Region	Dogs examined			Dogs infested		Fleas collected, *N* =														
						*C. canis*			*C. orientis*			*P. irritans*			Unidentifiable			Total		
	M	F	T	*N* =	%	M	F	T	M	F	T	M	F	T	M	F	T	M	F	T
Jizzax	14	10	24	12	50	6	13	19	1	12	13	0	0	0	0	2	2	7	27	34
Buxoro	7	5	12	7	58.3	6	23	29	2	2	4	0	0	0	0	1	1	8	26	34
Farg’ona	11	8	19	12	63.1	11	37	48	3	22	25	0	0	0	0	1	1	14	60	74
Samarkand	7	13	20	10	50	3	6	9	2	9	11	2	20	22	1	3	4	8	38	46
Surkxandaryo	0	2	2	2	100	1	9	10	0	0	0	0	0	0	0	1	1	1	10	11
Total	39	38	77	43	NA	27	88	115	8	45	53	2	20	22	1	8	9	38	161	199

## Discussion

Present Uzbekistan lies on the ancient trade route that linked the Western world with the Middle East and Asia called The Silk Road. This road connected the worlds for over 1500 years, and recent evidence suggests that cat domestication might have taken place along the route [27]. Domestication of dogs and cats has been speculated to be the reason behind the success of the cat flea domination on our household pets [8]. Countries in Central Asia (Kazakhstan, Kyrgyzstan, Tajikistan, Turkmenistan, Uzbekistan) may be particularly valuable in addressing this hypothesis and tracing the genetic signatures of fleas on domestic animals along the ancient Silk Road. Presence of *C. orientis* in Uzbekistan suggests that this species may have been introduced from the east along the ancient Silk Road, because *C. orientis* is commonly known from South Asia, East Asia and Southeast Asia [8].

In this study that focused on owned dogs, we demonstrate that *C. canis* is the dominant flea infesting dogs in Uzbekistan, followed by *C. orientis* and *P. irritans*. To the best of our knowledge, up to now, there are no studies on flea species infesting dogs in the other countries of Central Asia (Kazakhstan, Kyrgyzstan, Tajikistan, Turkmenistan). In Afghanistan, a geographically close country, two species of fleas parasitic in stray dogs were identified, *C. canis* and *P. irritans* [28], the first being dominant as in the present study. The identification and differentiation of *C. orientis* require mounting of fleas and precise observation of setae bearing notches on the hind tibia in combination with micro-setae above the antennal fossa together with further morphological features summarized in Lawrence et al. [8] and in the original descriptions [24, 29]. Secondary description and identification keys often are often based on European and North American veterinary literature and disregard *C. orientis*. Similarly, the previous recognition of the species *C. orientis* as a subspecies of *C. felis* renders the reliance on past literature for the distribution of *Ctenocephalides* species in Asia unreliable. This study where no *C. felis* was documented shows that traditional morphological identification of *C. orientis* can erroneously include 31–57% *C. canis* specimens. Even morphological identification of *C. canis* was incorrect in 6–9%. Traditionally, the differentiation relies on characters that in this case were variable. Besides the key presence or absence of characters and numerical features such as number of setae, subjectively the curvature of the cephalic profile has been used to discriminate *C. orientis* as *C. canis*. To remove the subjectivity, we employed geometric morphometrics to the shape or curvature of the head together with key head landmarks. Geometric morphometrics is a collection of approaches that provide a mathematical description of forms according to geometric definitions of their shape that transformed physical anthropology and other fields of biological sciences [30]. It is now argued that many so-called ‘cryptic species’ are not cryptic if analyzed using geometric morphometrics [31–35]. In our case with *C. canis* and *C. felis*, geometric morphometrics confirmed significant geometric differences in shape between these two species and their sexes. In addition, applying geometric morphometrics of only the flea head enabled us to improve by approximately four- to fivefold the species identification over the traditional morphological key.

Regional collections series and expert morphological characterization coupled with molecular confirmation based on *cox*1 provide the most reliable approach established for *Ctenocephalides* spp. [8]. The study by Lawrence et al. [8] was based on morphology and *cox*1 516 *Ctenocephalides* fleas across 56 countries; nevertheless, under-sampled areas of the world such as Central Asia, parts of the Middle East and Africa need further scrutiny [36–38]. This study from Uzbekistan added 166 *Ctenocephalides* flea *cox*1 sequences, thus increasing by one-third the sampling in Lawrence et al. [8]. In fact, Lawrence et al. [8] included only 58 *C. canis cox1* sequences compared to 115 in this study from Uzbekistan. Our phylogenetic analysis of *C. canis cox*1 sequences demonstrates that this species is much less diverse (based on Kimura-2 distance) than *C. felis*. The reason for such difference may lie in the generalist nature of *C. felis* infecting at least 130 wildlife species of 20% of all mammal species sampled for fleas while *C. canis* have been recorded only on 31 mammal species, mostly a narrow spectrum of canids, felids and murids [7].

In other studies, on dogs, *C. canis* was also the most prevalent flea species in Greece [39], Ireland [40], Argentina [41] and Korea [15]. The authors are aware that the number of examined dogs included in this study is not enough to have an overall picture of the actual distribution of *Ctenocephalides* fleas in Uzbekistan and that all the tested animals originated in the southeastern part of the country. The absence of studies in Central Asia together with the results of the present study calls for further epidemiological surveys of flea species in both domestic and wild carnivores from this region. Our results support the opinion that *C. canis* prefers colder climates than *C. felis*, and it is a species restricted to temperate zones [8, 14, 15, 42]. As we tested only dogs kept outside, we could not compare the association of flea species with the dog housing conditions (inside/outside; rural/urban area), but a positive correlation between dogs kept outside and the infestation with the dog flea was previously reported [15, 42], and it is in correlation with the present results.

It is not clear which is the actual reason for the absence of infestation with *C. felis*, but similar results were obtained in South Korea [15] and confirmed by the characterization of *cox1* from fleas on domestic dogs from South Korea that all belonged only to *C. canis* [8]. There are several potential factors that could contribute to the lack of this species in examined dogs, such as a very low prevalence of this species in dogs, considering the small number of examined animals, or the absence of examined dogs kept inside as pets, the season when the fleas were collected (winter) or even the continental climate in Samarkand region. Interestingly, *C. felis* was previously identified in domestic dogs from Uzbekistan [43]. In addition, in the same study, the authors did not report any infestation with *C. orientis*. Based on these and the lack of photos or complementary identification methods, we cannot exclude a misidentification of the fleas’ species in the only study previously done in Uzbekistan [43].

The second most frequent species, the oriental dog flea (*C. orientis*), is restricted to Asia [8], and our study represents the first report of *C. orientis* in Central Asia. The species prefers tropical and subtropical zones and has a wide host spectrum, more commonly identified in ruminants than in dogs and cats [8, 12, 17]. Out of the five examined areas, the species was present only in four. Its absence in Surkxandaryo region is likely related to the low number of examined animals from that area or the higher altitude (Fig. 4). The exact geographical distribution of this species is not currently known, but there are some hypotheses that climate change and livestock movements could contribute to the spreading of this species [44] or even be a relic from the ancient times when the Silk Road connected Asia with the Mediterranean.

*Pulex irritans*, the human flea, were identified in dogs from a single region. Being a species that can infest a wide range of hosts, its absence in other geographical locations can be related to its host preferences, seasonality or climatic preferences [14]. Finding *P. irritans* in dogs is not common but at the same time not unexpected, as it has been found in other regions of the world, e.g. [45].

## Conclusions

This study aimed to investigate the flea infestation in privately owned dogs kept outdoors in five locations in Uzbekistan. The most common flea species infesting dogs is *C. canis* followed by *C. orientis* and *P. irritans*. No dogs were infested with *C. felis*. We report for the first time the presence of *C. orientis* in a country in Central Asia, Uzbekistan, using a robust and reproducible methodology that employed both *cox1* DNA sequencing and geometric morphometrics. A landmark-based geometric morphometric analysis of flea heads is a useful additional method for species discrimination. This study highlights the ambiguity of identification of *Ctenocephalides* spp. when *C. orientis* is present based on traditional morphological features. Studies across Asia are needed to expose the actual distribution of *C. orientis* to improve our understanding of the role of the Silk Road connecting Asia with the Mediterranean played in parasite dispersal.

## Supplementary Information

**Additional file 1: Table S1.** Table showing the prevalence of infested dogs. **Table S2**. The number of examined and positive dogs regarding their sex. **Table S3.** The number of examined and positive dogs regarding their age. **Table S4**. The number of dogs infested with *C. canis* from each of the locations. **Table S5**. The number of dogs infested with *C. orientis* from each of the locations. **Table S6. **The number of dogs infested with *P. irritans* from each of the locations. **Table S7**. The number of dogs infested with unidentified fleas from each of the locations. **Table S8**. Number of infested dogs in each location. **Table S9**. Species of fleas for each of the age groups. **Table S10**. The sex of the identified fleas.

## Data Availability

Details about the tested dogs and identified fleas are available in the additional file. The nucleotide sequence data generated in this study were deposited in GenBank (NCBI) under the accession numbers ON247050-ON247215. Sequence comparative data and the associated additional data including geometric morphometrics (images, MorphoJ, tps) are available at LabArchives (https://dx.doi.org/10.25833/c9qj-ha67). The mounted fleas are preserved in the collection of the Department of Parasitology and Parasitic Diseases from USAMV Cluj-Napoca Romania and are available from the first author on reasonable request.

## References

[1] Pilgrim RLC (1991). Fleas. NZ Entomol.

[2] Whitaker AP (2007). Fleas: siphonaptera.

[3] Márquez FJ, Millán J, Rodriguez-Liebana JJ, Garcia-Egea I, Muniain MA (2009). Detection and identification of *Bartonella* sp. in fleas from carnivorous mammals in Andalusia, Spain. Med Vet Entomol.

[4] Krämer F, Mencke N (2001). Flea biology and control.

[5] Blagburn BL, Dryden MW (2009). Biology, treatment, and control of flea and tick infestations. Vet Clin North Am Small Anim.

[6] Kernif T, Socolovschi C, Wells K, Lakim MB, Inthalad S, Slesak G (2012). *Bartonella* and *Rickettsia* in arthropods from the Lao PDR and from Borneo, Malaysia. Comp Immunol Microbiol Inf Dis.

[7] Clark NJ, Seddon JM, Šlapeta J, Wells K (2018). Parasite spread at the domestic animal-wildlife interface: anthropogenic habitat use, phylogeny and body mass drive risk of cat and dog flea (*Ctenocephalides* spp.) infestation in wild mammals. Parasit Vectors.

[8] Lawrence AL, Webb CE, Clark NJ, Halajian A, Mihalca AD, Miret J (2019). Out-of-Africa, human-mediated dispersal of the common cat flea, *Ctenocephalides felis*: the hitchhiker’s guide to world domination. Int J Parasitol.

[9] Just FT, Gilles J, Pradel I, Pfalzer S, Lengauer H, Hellmann K (2008). Molecular evidence for *Bartonella* spp. in cat and dog fleas from Germany and France. Zoonoses Public Health..

[10] Giudice E, Di Pietro S, Alaimo A, Blanda V, Lelli R, Francaviglia F (2014). A molecular survey of *Rickettsia felis* in fleas from cats and dogs in Sicily (Southern Italy). PLoS ONE.

[11] Lawrence AL, Brown GK, Peters B, Spielman DS, Morin-Adeline V, Šlapeta J (2014). High phylogenetic diversity of the cat flea (*Ctenocephalides felis*) at two mitochondrial DNA markers. Med Vet Entomol.

[12] Colella V, Nguyen VL, Tan DY, Lu N, Fang F, Zhijuan Y (2020). Zoonotic vectorborne pathogens and ectoparasites of dogs and cats in Eastern and Southeast Asia. Emerg Infect Dis.

[13] Zurita A, Benkacimi L, El Karkouri K, Cutillas C, Parola P, Laroche M (2021). New records of bacteria in different species of fleas from France and Spain. Comp Immunol Microbiol Inf Dis.

[14] Beugnet F, Labuschagne M, Fourie J, Jacques G, Farkas R, Cozma V (2014). Occurrence of *Dipylidium caninum* in fleas from client-owned cats and dogs in Europe using a new PCR detection assay. Vet Parasitol.

[15] Ahn KS, Huh SE, Seol SW, Kim H, Suh KH, Shin S (2018). *Ctenocephalides canis* is the dominant flea species of dogs in the Republic of Korea. Parasit Vectors.

[16] Hii SF, Lawrence AL, Cuttell L, Tynas R, Abd Rani PAM, Šlapeta J (2015). Evidence for a specific host-endosymbiont relationship between ‘*Rickettsia* sp. genotype RF2125’and *Ctenocephalides felis* orientis infesting dogs in India. Parasit Vectors.

[17] Ashwini M, Puttalakshmamma G, Mamatha G, Chandra Naik B, Thimmareddy P (2017). In-vitro studies on biology of *Ctenocephalides orientis* fleas infesting sheep and goat. J Entomol Zool Stud.

[18] Ioff I, Efremova N. The Biology and Fauna of Fleas on Domestic Animals in Central Asia. 1927; 3–4.

[19] Safarov AA, Azimov DA, Akramova FD (2018). Taxonomical structure of dogs’ population ectoparasites (*Canis familiaris* dom.) in Tashkent megapolis Uzbekistan. Eur Sci Rev.

[20] Shernazarov ES, Vashetko EV, Kreitsberg EA. Vertebrates of Uzbekistan. Tashkent [Russian language]. 2006

[21] Yong TS, Lee KJ, Shin MH, Yu HS, Suvonkulov U, Sergeevich TB (2009). Prevalence of intestinal helminth infections in dogs and two species of wild animals from Samarkand Region of Uzbekistan. Korean J Parasitol.

[22] Safarov AA, Norqobilov BT, Azimov DA, Akramova FD, Mavlonov SI (2021). The parasites of dogs in Tashkent metropolis.

[23] Whiting MF, Whiting AS, Hastriter MW, Dittmar K (2008). A molecular phylogeny of fleas (Insecta: Siphonaptera): origins and host associations. Cladistics.

[24] Hopkins GHE, Rothschild M, Rothschild M, Mardon DK (1953). An illustrated catalogue of the rothschild collection of fleas (Siphonaptera) in the British Museum (Natural History) with keys and short descriptions for the identification of families, genera, species and subspecies. Tungidae and Pulicidae.

[25] Klingenberg CP (2011). MorphoJ: an integrated software package for geometric morphometrics. Molec Ecol Res.

[26] Tamura K, Stecher G, Kumar S (2021). MEGA11: molecular evolutionary genetics analysis version 11. Mol Biol Evol.

[27] Haruda AF, Ventresca Miller AR, Paijmans JLA, Barlow A, Tazhekeyev A, Bilalov S (2020). The earliest domestic cat on the Silk Road. Sci Rep.

[28] Le Riche PD, Soe AK, Alemzada Q, Sharifi L (1988). Parasites of dogs in Kabul. Afghanistan Br Vet J.

[29] Jordan K (1925). New Siphonaptera. Novit Zool.

[30] Slice DE (2007). Geometric morphometrics. Annu Rev Anthropol.

[31] Karanovic T, Djurakic M, Eberhard SM (2016). Cryptic species or inadequate taxonomy? Implementation of 2D geometric morphometrics based on integumental organs as landmarks for delimitation and description of copepod taxa. Syst Biol.

[32] Chatpiyaphat K, Sumruayphol S, Dujardin JP, Samung Y, Phayakkaphon A, Cui L (2021). Geometric morphometrics to distinguish the cryptic species *Anopheles minimus* and *An. harrisoni* in malaria hot spot villages, western Thailand. Med Vet Entomol.

[33] Sontigun N, Sukontason KL, Zajac BK, Zehner R, Sukontason K (2017). Wing morphometrics as a tool in species identification of forensically important blow flies of Thailand. Parasit Vectors.

[34] Rodríguez-González A, Míguez-Lozano R, Llopis-Belenguer C, Balbuena JA (2015). Phenotypic plasticity in haptoral structures of *Ligophorus cephali* (Monogenea: Dactylogyridae) on the flathead mullet (*Mugil cephalus*): a geometric morphometric approach. Int J Parasitol.

[35] Bakkes DK, Chitimia-Dobler L, Matloa D, Oosthuysen M, Mumcuoglu KY (2020). Integrative taxonomy and species delimitation of *Rhipicephalus turanicus* (Acari: Ixodida: Ixodidae). Int J Parasitol.

[36] Power RI, Calvani NED, Nachum-Biala Y, Salant H, Harrus S, Šlapeta J (2021). Adaptation of gltA and ssrA assays for diversity profiling by Illumina sequencing to identify *Bartonella henselae*, *B. clarridgeiae* and *B. koehlerae*. J Med Micr.

[37] Van der Mescht L, Matthee S, Matthee CA (2021). New taxonomic and evolutionary insights relevant to the cat flea, *Ctenocephalides felis*: a geographic perspective. Mol Phylogen Evol.

[38] Calvani NE, Bell L, Carney A, De La Fuente C, Stragliotto T, Tunstall M (2020). The molecular identity of fleas (Siphonaptera) carrying *Rickettsia felis*, *Bartonella clarridgeiae* and *Bartonella rochalimae* from dogs and cats in Northern Laos. Heliyon.

[39] Koutinas AF, Papazahariadou MG, Rallis TS, Tzivara NH, Himonas CA (1995). Flea species from dogs and cats in northern Greece: environmental and clinical implications. Vet Parasitol.

[40] Wall R, Shaw SE, Penaliggon J (1997). The prevalence of flea species on cats and dogs in Ireland. Med Vet Entomol.

[41] González A, del Castro DC, González S (2004). Ectoparasitic species from *Canis familiaris* (Linné) in Buenos Aires province, Argentina. Vet Parasitol..

[42] Franc M, Choquart P, Cadiergues MC (1998). Répartition des espèces de puces rencontrées chez le chien en France. Rev Med Vet.

[43] O’G’LI SA, Safarov AA, Azimovich AJ, Jaloliddinovna AF (2018). Taxonomical structure of dogs’ population ectoparasites (*Canis familiaris* dom.) in Tashkent megapolis, Uzbekistan. Eur Sci Rev.

[44] Seyyed-Zadeh SJ, Bozorg-Omid F, Telmadarraiy Z, Terenius O, Chavshin AR (2018). Evidence for the presence of *Ctenocephalides orientis* in livestock dwellings in northwest Iran. Med Vet Entomol.

[45] Chandra S, Forsyth M, Lawrence AL, Emery D, Šlapeta J (2017). Cat fleas (*Ctenocephalides felis*) from cats and dogs in New Zealand: molecular characterisation, presence of *Rickettsia felis* and *Bartonella clarridgeiae* and comparison with Australia. Vet Parasitol.

